# A novel approach to study multi-domain motions in JAK1’s activation mechanism based on energy landscape

**DOI:** 10.1093/bib/bbae079

**Published:** 2024-03-05

**Authors:** Shengjie Sun, Georgialina Rodriguez, Gaoshu Zhao, Jason E Sanchez, Wenhan Guo, Dan Du, Omar J Rodriguez Moncivais, Dehua Hu, Jing Liu, Robert Arthur Kirken, Lin Li

**Affiliations:** Department of Biomedical Informatic, School of Life Sciences, Central South University, Changsha 410083, China; Computational Science Program, The University of Texas at El Paso, 500 W University Ave, TX 79968, USA; Department of Biological Sciences, The University of Texas at El Paso, 500 W University Ave, TX 79968, USA; Border Biomedical Research Center, The University of Texas at El Paso, 500 W University Ave, TX, 79968, USA; Google LLC, 1600 Amphitheatre Parkway Mountain View, CA 94043, USA; Computational Science Program, The University of Texas at El Paso, 500 W University Ave, TX 79968, USA; Computational Science Program, The University of Texas at El Paso, 500 W University Ave, TX 79968, USA; Computational Science Program, The University of Texas at El Paso, 500 W University Ave, TX 79968, USA; Department of Biological Sciences, The University of Texas at El Paso, 500 W University Ave, TX 79968, USA; Border Biomedical Research Center, The University of Texas at El Paso, 500 W University Ave, TX, 79968, USA; Department of Biomedical Informatic, School of Life Sciences, Central South University, Changsha 410083, China; Department of Hematology, The Second Xiangya Hospital of Central South University; Molecular Biology Research Center, Center for Medical Genetics, School of Life Sciences, Central South University, Changsha 410083, China; Department of Biological Sciences, The University of Texas at El Paso, 500 W University Ave, TX 79968, USA; Border Biomedical Research Center, The University of Texas at El Paso, 500 W University Ave, TX, 79968, USA; Computational Science Program, The University of Texas at El Paso, 500 W University Ave, TX 79968, USA; Google LLC, 1600 Amphitheatre Parkway Mountain View, CA 94043, USA; Department of Physics, The University of Texas at El Paso, 500 W University Ave, TX 79968, USA

**Keywords:** JAK1, tyrosine kinase, Dijkstra’s method, delphi, electrostatic potential, energy landscape

## Abstract

The family of Janus Kinases (JAKs) associated with the JAK–signal transducers and activators of transcription signaling pathway plays a vital role in the regulation of various cellular processes. The conformational change of JAKs is the fundamental steps for activation, affecting multiple intracellular signaling pathways. However, the transitional process from inactive to active kinase is still a mystery. This study is aimed at investigating the electrostatic properties and transitional states of JAK1 to a fully activation to a catalytically active enzyme. To achieve this goal, structures of the inhibited/activated full-length JAK1 were modelled and the energies of JAK1 with Tyrosine Kinase (TK) domain at different positions were calculated, and Dijkstra’s method was applied to find the energetically smoothest path. Through a comparison of the energetically smoothest paths of kinase inactivating P733L and S703I mutations, an evaluation of the reasons why these mutations lead to negative or positive regulation of JAK1 are provided. Our energy analysis suggests that activation of JAK1 is thermodynamically spontaneous, with the inhibition resulting from an energy barrier at the initial steps of activation, specifically the release of the TK domain from the inhibited Four-point-one, Ezrin, Radixin, Moesin-PK cavity. Overall, this work provides insights into the potential pathway for TK translocation and the activation mechanism of JAK1.

## Graphical Abstract

**Graphical Abstract:**
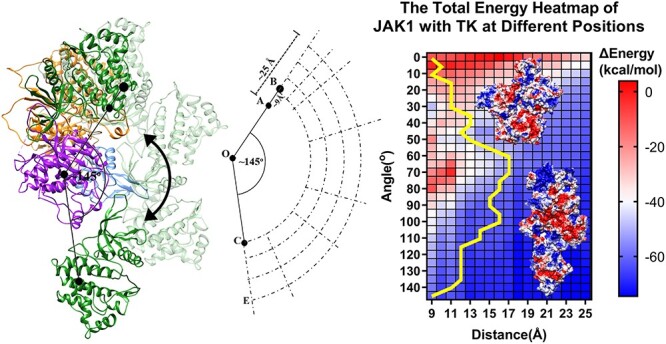

## INTRODUCTION

Cytokine receptor associated Janus kinases (JAKs) play a crucial role in the regulation of various cellular processes, including immune and inflammatory responses [1], hematopoiesis [2, 3] and oncogenesis [4]. JAK family members, including JAK1, JAK2, JAK3 and TYK2, are noncovalently bound to the intracellular domain of Type I and Type II cytokine receptors. When small molecule cytokines bind to the extracellular domains of cytokine receptors it leads to dimerization of receptor subunits, triggering associated JAK proteins to autophosphorylate tyrosine residues within the tyrosine kinase (TK) domain [5, 6]. The intracellular domains of receptor subunits serve as the binding sites for signal transducers and activators of transcription (STATs) which JAKs activate through tyrosine phosphorylation [7]. STATs are further phosphorylated by serine/threonine kinases [8], dimerize [9] and translocate to the nucleus where they regulate gene transcription. Collectively, the process from JAK autoactivation to initiation of transcription in the nucleus by STATs is known as the JAK–STAT signaling pathway [10].

Structurally JAKs are comprised of seven Janus homology (JH) domains (Figure S1) and include a Four-point-one, Ezrin, Radixin, Moesin (FERM) domain (JH5, JH6, and JH7), an Src Homology 2 (SH2) domain (JH3 and JH4), and two kinase domains (JH2 and JH1). The JH2 region, a Pseudokinase (PK) domain is non-catalytic but is capable of binding ATP and tightly regulates kinase activity. [11]. The FERM and SH2 domains are tightly associated with juxta-membrane domain cytokine receptor [12–14]. Specifically, PK has autoinhibitory domains that keep TK inactive until stimulation from cytokine receptor dimerization [15]. A classic example that illustrates this feature is the JAK1 Val-to-Phe (VF) mutation of PK residue 657. The homologous JAK2 V617F is the most common mutation in JAK2-PK, which results in constitutive activity [16]. The V617F mutation causes simple steric disruption, disturbing the inhibited state of TK to activate JAK2 [14, 17]. Notably, in the absence of receptor dimerization, the activated state is likely transient. JAK1 V657F mutation presumably promotes JAK1 dimer formation [18], freeing the TK for constitutive activity and continuous transphosphorylation [19]. These findings reveal that the PK domain of JAKs have two functions: firstly, to bind TK keeping it in an autoinhibited state and preventing its release; secondly, to stabilize an active JAK dimer once formed. The P733L mutation, which also occurs within the JAK1-PK domain, results in impaired transphosphorylation related to immunodeficiency [20]. Besides, H595D and A634D mutation can cause autoinflammation, immune dysregulation, and eosinophilia [21]. Both mutation sites are located on the PK. In contrast, S703I is a JAK1 PK gain-of-function mutation found to increase JAK1 dimerization and kinase activity leading to hyper cytokine.

**Figure 1:**
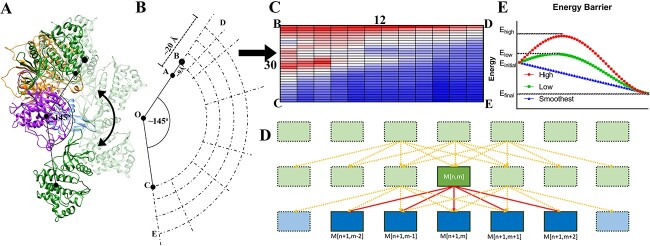
The transitional activation process of JAK1 according to the path analysis method. (**A** and **B**) Diagram of the activation–inhibition process. Knot O represents the mass center of PK. Knot A and C represents the mass centers of TK in inhibited and activated states, respectively. B is the mass center of TK after separating TK from PK by 9 Å to make OC=OB. D represents the mass center of TK for 25 Å separation while E represents the corresponding mass center of TK after a 145^o^ rotation from position D. (**C**) The heatmap represents the total energy matrix of JAK1 protein when TK domain is at different positions obtained by the transformations outlined in b. (**D**) The possible position of TK after each rotation. (**E**) Two simulated paths with high/low energy barrier and the smoothest path with no energy barrier.

To fully understand the pathogenic mechanism of PK domain mutations it is necessary to discern the transitional JAK activation process. However, simulating the entire process using all-atom explicit Molecular Dynamics (MD) simulations is challenging due to resource requirements and unknown full-length inhibited JAK structures. Previous reports focused on JH1 domain bound to inhibitors to enhance computational resource efficiency and minimize wastage [22, 23]. These works using MD simulations resolved the structural-molecular interactions of bound JAKs inhibitors to validate JH1-inhibitor docking results. However, they did not address the JAKs activation process, which remains unresolved. In 2014, an autoinhibited structure of the JAK PK-TK dimer was revealed [15] and more recently the full-length mouse JAK1 homo-dimer structure was established by cryo-electron microscopy, providing an opportunity to construct the full-length JAK1 in its inhibited state [19]. An energetic and steric analysis should reveal potential paths of TK transition from inhibited (closed) to activated (open) states. In this study we aligned two PK monomers, one from an inhibited PK-TK dimer [15] and the other from an activated full-length JAK1 [19] to determine the position of TK in an inhibited form and construct the full-length inhibited JAK1 structure. Interestingly, the interfaces of TK domains face the PK domains in both inhibited and activated states identically. This finding suggests a high likelihood that TK rotates around PK to reach the activated position (Figure 1**A**). However, the TK domain in an activated state is slightly far from PK compared to inhibited states further suggesting rotation and separation contributes to TK activation. Based on separation and rotational movement we modeled a TK transition around PK to build an energy map and find the smoothest path for TK activation. Each dot in the energy map represents a knot, indicating that the number of possible paths exceeds n^m^ (where n and m represent the rotation degree and separation distances, respectively). Therefore, computational methods [24] are required to reduce the complexity of these paths. Based on Dijkstra’s algorithm [25, 26], we have developed a novel algorithm that builds upon Dijkstra’s method to identify the path of minimal variance, which represents the smoothest transition from an inactive to an active kinase state. This innovative approach can be applied not only to kinase proteins but also to other biomolecules that consist of multiple domains.

## METHODS

### Modeling and molecular dynamics simulations

The full-length activated (open) and inhibited (closed) human JAK1 structures were generated using SWISS-MODEL [27] based on activated the JAK1 dimer (PDB: 7T6F; resolution 3.60 Å) [19] and inhibited JH1-JH2 dimer (PDB: 4OLI; resolution 2.80 Å) [15, 28]. The RMSD between the activated JAK1 model and the template (PDB: 7T6F) is 0.10 Å while the RMSD between inhibited JAK1 model and the template (PDB: 4OLI) is 0.40 Å. The JAK1 sequence used for modeling is the *Homo sapiens* tyrosine-protein kinase JAK1 sequence, which can be obtained from UniProt (P23458) [29]. It has a 94.72% identity with the activated JAK1 dimer template 7T6F (Query Cover: 97%) and 54.97% identity with inhibited PK-TK dimer template 4OLI (Query Cover: 91%). Models were then solvated with TIP3P [30] and ionized by 150 mM KCl via CHARMM-GUI [31]. The force field applied in MD simulations is CHARMM36m [32], which is with improved accuracy in generating polypeptide backbone and proteins. Periodic boundary conditions and the long-range electrostatic interactions with particle mesh Ewald [33] were performed during simulations. The temperature was set at 310.15 K, using a Langevin thermostat with a damping coefficient of 1/ps. The pressure was set to 1 atm using a Nosé–Hoover Langevin piston barostat with a decay period of 25 fs. Simulations were performed after 10 000-step minimization. Simulation included two steps: NPT equilibrium and NVT production. In the equilibrium phase, the backbones of proteins were restrained. In production, all atoms were freed. The simulations were performed by NAMD 2.12 [34]. The 0.5 ns NPT equilibrium and 50 ns NVT production were performed for each MD simulation (Table S1).

### Electrostatic analysis

The electrostatic potential was calculated by Delphi [35–39], which calculates the electrostatic potential ϕ by solving the Poisson–Boltzmann equation (Eq. 1) using finite difference method. The electrostatic force was further derived from electrostatic potential and charges by DelphiForce [40]. The charge and radius were assigned by pdb2pqr [41] from CHARMM36m [32]. The dielectric constant for protein and water were set to 2 and 80, respectively. The electrostatic force on the TK was calculated by DelphiForce [42, 43] (Figure S4). 


(1) \begin{equation*} \nabla \cdot \left[\mathrm{\varepsilon} \left(\mathrm{r}\right)\nabla \mathrm{\phi} \left(\mathrm{r}\right)\right]=-4\mathrm{\pi} \mathrm{\rho} \left(\mathrm{r}\right)+\mathrm{\varepsilon} \left(\mathrm{r}\right){\mathrm{\kappa}}^2\left(\mathrm{r}\right)\sinh \left(\mathrm{{\Phi}}\left(\mathrm{r}\right)/{k}_BT\right) \end{equation*}

where $\mathrm{\phi}$(r) and ρ(r) represents the electrostatic potential and the permanent charge density, ε(r) is the dielectric permittivity, *κ* is the Debye–Hückel parameter, *k_B_* is the Boltzmann constant, and *T* is temperature.

Salt concentration, probe radius, filling ratio of protein, and resolution were set to 150 mM, 1.4 Å, 0.70 and 2 grid/Å, respectively. The hydrogen bond analyses were performed on visual molecular dynamics (VMD) based on the last 10 ns MD simulations. The cutoff distance and the angle for hydrogen bond analysis were set to 3.5 Å and 20°, respectively.

### Simulation of kinase activation and analysis of the smoothest path

#### Simulation of kinase activation

Here we separate the JAK1 into four domains (FERM: residues 34–420, SH2: residues 439–544, PK: residues 583–855, and TK: residues 875–1153) and rotate the TK about the surface of PK to simulate the activation process (Figure 1**A**). The three mass centers of PK, TK in the inhibited state (TK-I), and TK in the activated state (TK-A) were used to define a plane. The plane was applied for the rotation and separation of TK in JAK1 activation.

TK predominantly behaves as a rigid body (RMSD: 1.195 Å) during the reorganization except very minor adjustments. First, TK and PK were separated by 25 Å. For each 1 Å increment, 1000 steps of minimization were applied. TK was then rotated along PK at 5° increments (totaling 145°) for each separation. Similarly, for each of these rotations, 1000 steps of minimization were applied. The grid defining this procedure is shown in Figure 1**B**. Here, the separation and rotation were achieved by StructureMan [44] while the minimization was applied by NAMD2.12 [34] with the CHARMM36m force field [32]. After each 1000-step minimization, the total energy (sum of the potential energies) was calculated by NAMD and used to construct the total energy matrix (M^30 x 17^) Figure 1**C**.

#### Analysis of the smoothest path

After testing at different separation sites, we can draw the energy map of JAK1 with TK located at different positions, and then calculate the possible path for activation. An energy matrix_30X17_ (M_30 × 17_) (Figure 1**C**) is built based on the separation between 9 to 25 Å and the rotation from 0 ^o^ to 145 ^o^, where rows represent the rotation degree while columns represent the separation distance. We are looking for a path from M [1] (After 9 Å separation of TK from the inhibited state) to M [1, 30] (activated state). The total energy change from M [1] to M [1, 30] is a constant, which is independent from the path. The minimal energy consumption cannot be a criterion to optimize the path. Therefore, we tried to find the smoothest path for TK to move from M [1] to M [1, 30]. The smoothest path means the energy consumption is even among steps during the activation process. Each step represents the TK rotation for 5^o^ in our simulations. Here we used the variance of the differences between the two steps to represent the level of smoothness (Eq. 2).


(2) \begin{equation*} {\sigma}^2=\frac{\sum_{i=1}^n{\left({\varDelta E}_{i,i+1}-\overline{\varDelta E}\right)}^2}{n-1} \end{equation*}

where the ${\sigma}^2$ represents the variance, ${\varDelta E}_{i,i+1}$ represents the energy differences between ${i}^{th}$ position and ${\left(i+1\right)}^{th}$ position. n equal to 29, because of the rotation is via each 5^o^ rotation/step for 145 ^o^ rotations to reach the activated state. $\overline{\varDelta E}$ represents the mean of all differences (Eq. 3).


(3) \begin{equation*} \overline{\varDelta E}=\frac{\sum_{i=1}^n\left({\varDelta E}_{i,i+1}\right)}{n}=\frac{\sum_{i=1}^n\left({E}_{i+1}-{E}_i\right)}{n}=\frac{\left({E}_{30}-{E}_1\right)}{n} \end{equation*}

where the ${E}_i$ represents the energy at ${i}^{th}$ position. ${E}_1$ is the value of M [1] and ${E}_{30}$ is the value of M [1, 30]. Because $\overline{\varDelta E}$ is a constant, equation 2 simplifies to equation 4.


(4) \begin{equation*} {\sigma}^2=\frac{\sum_{i=1}^n{{\varDelta E}_i}^2}{28}+a\sum_{i=1}^n{\varDelta E}_i+b \end{equation*}

where a and b are constants. Since the ${\varDelta E}_i$ is the solo contributor to variance, if use ${\left({\varDelta E}_i-\overline{\varDelta E}\right)}^2$ as the ‘distance’ of steps, the variance represents the length of the path and the shortest path can be easily solved by Dijkstra’s Method [45]. For each step (rotation), TK can move slightly closer or farther from PK. Here, we set each step to follow two rules:

(i) M_[n,m]_ can reach M_[n + 1,(m-2,m + 3)]_, (n < 29, m ≤17) (Figure 1**D**).

(ii) The initial position is M [1] and the last position should be M [1, 30].

Based on Eq. 4, the value of $\sum_{i=1}^n{{\varDelta E}_i}^2$ is only contributor to ${\sigma}^2$. By replacing ${y}_i$ to 1 in Cauchy–Schwarz inequality


(5) \begin{equation*} {\left(\sum{x}_i{y}_i\right)}^2\le \left(\sum{x_i}^2\right)\left(\sum{y_i}^2\right) \end{equation*}

we can get the minimal ${\sigma}^2$


(6) \begin{equation*} {\sigma}^2=a{\left(\sum_{i=1}^n{\varDelta E}_i\right)}^2+c \end{equation*}

In Figure 1**E**, we simulated several paths of high/low energy barrier or smoothest path. For the high energy barrier path, the ${E}_h=\left({\sum}_{i=1}^n{\varDelta E}_i\right)$ is equal to (${E}_{high}-{\varDelta E}_{initial}$)+$\left({E}_{high}-{E}_{final}\right)=2\left({E}_{initial}-{E}_{final}\right)+\left({E}_{high}-{\varDelta E}_{initial}\right)$.

For the low energy barrier path, the ${E}_l=\left({\sum}_{i=1}^n{\varDelta E}_i\right)$ is equal to (${E}_{low}-{\varDelta E}_{initial}$)+$\left({E}_{low}-{E}_{final}\right)=2\left({E}_{initial}-{E}_{final}\right)+\left({E}_{low}-{\varDelta E}_{initial}\right)$.

For the smoothest path, ${E}_s==\left({\sum}_{i=1}^n{\varDelta E}_i\right)$ is equal to $\left({E}_{initial}-{E}_{final}\right)$.

It’s clear that in ${E}_h>{E}_l>{E}_s$, our approach always chose the path with no energy barrier or the lowest energy barrier. Compared with traditional nudged elastic band technique [46], our method is far more suitable for macromolecules.

### Root Mean Square Fluctuation (RMSF) and Root Mean Square Deviation (RMSD)

The RMSF of the α-carbons of the residues (21 residues) on the TMD is achieved based on the last 40 ns simulations by VMD [47] (Eq. 5) [48].


(5a) \begin{equation*} {RMSF}_i={\left[\frac{1}{T}\sum_{t_j=1}^T|{r}_i\left({t}_j\right)-{r}_i^{ref}|\right]}^{1/2} \end{equation*}

Where $i$ represents the residue ID, the $T$ represents the total simulation time (Here is the number of frames), ${r}_i\left({t}_j\right)$ represents the residues $i$ in the time of ${t}_j$ position. The ${r}_i^{ref}$ is the reference position of residue $i$, calculated by the time-average position.

The root-mean-square deviation (RMSD) is to measure the average distance between two protein structures, calculated by equation 6 [47, 48].


(6a) \begin{equation*} RMSD(t)={\left[\frac{1}{WN}\sum_{i=1}^N{w}_i{\left|{r}_i(t)-{r}_i^{ref}\right|}^2\right]}^{1/2} \end{equation*}

Where $W$ = Σ${w}_i$ is the weighting factor, and $N$ is the total number of atoms. The ${r}_i(t)$ is the position of atom $i$ at time $t$ after least square fitting the structure to the reference structure. The ${r}_i^{ref}$ is the reference position of residue $i$ defined by the reference structure.

## RESULTS

### Intramolecular forces of the autoinhibited closed JAK1 monomer

**Figure 2:**
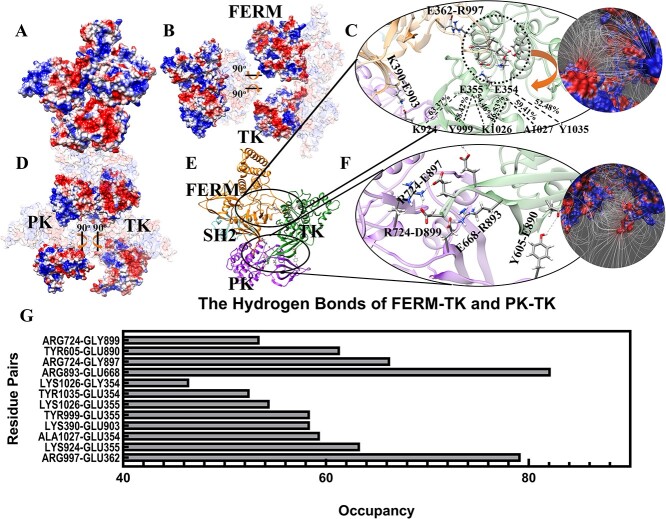
The electrostatic surface and hydrogen bonds of inhibited full-length JAK1 monomer. (**A**) The electrostatic surface of full-length JAK1 structure. (**B**) The electrostatic surface of the binding interface between FERM and TK (The interfaces of FERM and TK are rotated open 90^o^) to show the FERM-TK interaction. (**C**) The high-occupancy hydrogen bonds between FERM and TK. (**D**) The electrostatic surface of the binding interface between PK and TK (PK and TK are oppositely rotated 90^o^). (**E**) The structural representation and high-occupancy hydrogen bonds for inhibited full-length JAK1. (**F**) The high-occupancy hydrogen bonds between PK and TK. (**G**) The occupancies of hydrogen bonds of FERM-TK and PK-TK.

After 40 ns of MD simulations, the structure of the inhibited full-length JAK1 reached a stable state (Figure S2). The structures from the last 10 ns of the simulation (40–50 ns) were chosen for electrostatic analysis. The surface of inhibited full-length JAK1 has a pentagram-like shape (Figure 2**A**), where the TK is tightly bound by both FERM and PK. PK and FERM provide the main binding forces to stabilize the TK in the inhibited state (Figure S5). After separating and rotating FERM and TK domains by 90^o^, we observed the left and right surfaces of TK are negatively and positively charged, respectively. Correspondingly, the left end of FERM is positively charged while the middle right is negatively charged (Figure 2**B** and Figure S3). The electrostatic attraction between the FERM and TK domains likely plays a vital part in securing their binding. Additionally, an analysis of hydrogen bonds reveals the presence of many hydrogen bonds formed between the FERM and TK domains. Glutamic acid residues E354 (~160%) and E355 (~180%) contributed the most hydrogen bonding by interacting with K924, Y999, K1026, A1027 and Y1035 (Figure 2**C**). Notably, JAK1 residues Y1034/Y1035 of the TK activation loops are involved in regulation of kinase activity [49]. E354 is located in the fatty acid binding sites for Ruxolitinib [50], a JAK inhibitor used to treat myelofibrosis. Interaction between Ruxolitinib and E354 may prevent the release of TK, making it harder for the JAK to be activated. Additionally, E362 and K390 are also important residues with high occupancies (79.21% and 58.42%, respectively) of hydrogen bonds. These bonds play a significant role in strengthening the interaction between the two domains, further contributing to the stability of the protein and suggesting they could be the potential targets for inhibitor design.

The interactions between PK and TK domains are depicted in Figure 2**D**, where PK and TK have been rotated open by 90^o^ to display the distribution of electrostatic potential on the interaction interface. The right part of TK is negatively charged and the corresponding left part in PK is positively charged. The inhibition of TK can be explained, at least in part, by these electrostatic attractions. A deeper analysis of hydrogen bonds uncovered the contribution of residue pairs. PK residue R724 is the most significant contributor to hydrogen bonds, forming bonds with both E897 and D899. This observation has been previously noted in studies that examine the inhibited interfaces between the JAK2 PK-TK dimer (R744 in JAK2) [14]. PK residue E668 also makes a significant contribution to hydrogen bonding, with 82.18% occupancy in its interaction with R893. This phenomenon has been previously reported by other studies (E688 in JAK2, 14]. Notably, a new hydrogen bond was discovered by our MD simulations between PK residue Y605 and TK E890, with occupancy of 61.39%. Because tyrosine phosphorylation is a critical step for JAK activation [51–53], it is possible that Y605 phosphorylation maybe involved in JAK1 activation [54]. JAK1 Y605 is reported to be phosphorylated within the T cell leukemia Jurkat cell line treated with phosphatase inhibitor by the PhosphoSitePlus consortium. H595D and A634D have been reported in association with autoinflammation, immune dysregulation, and eosinophilia. Interestingly, these mutations are located within the protein kinase PK, rather than on the interface. It is hypothesized that these mutations may induce structural changes within the PK domain, potentially affecting the formation of hydrogen bonds at the interface between JAK1 and its interacting partners.

Intramolecular hydrogen bonds indicate the residue pair’s contribution to maintaining a closed state and imply the potential a mutation might have to change the structure.

### Intramolecular forces of the activated JAK1 monomer

**Figure 3:**
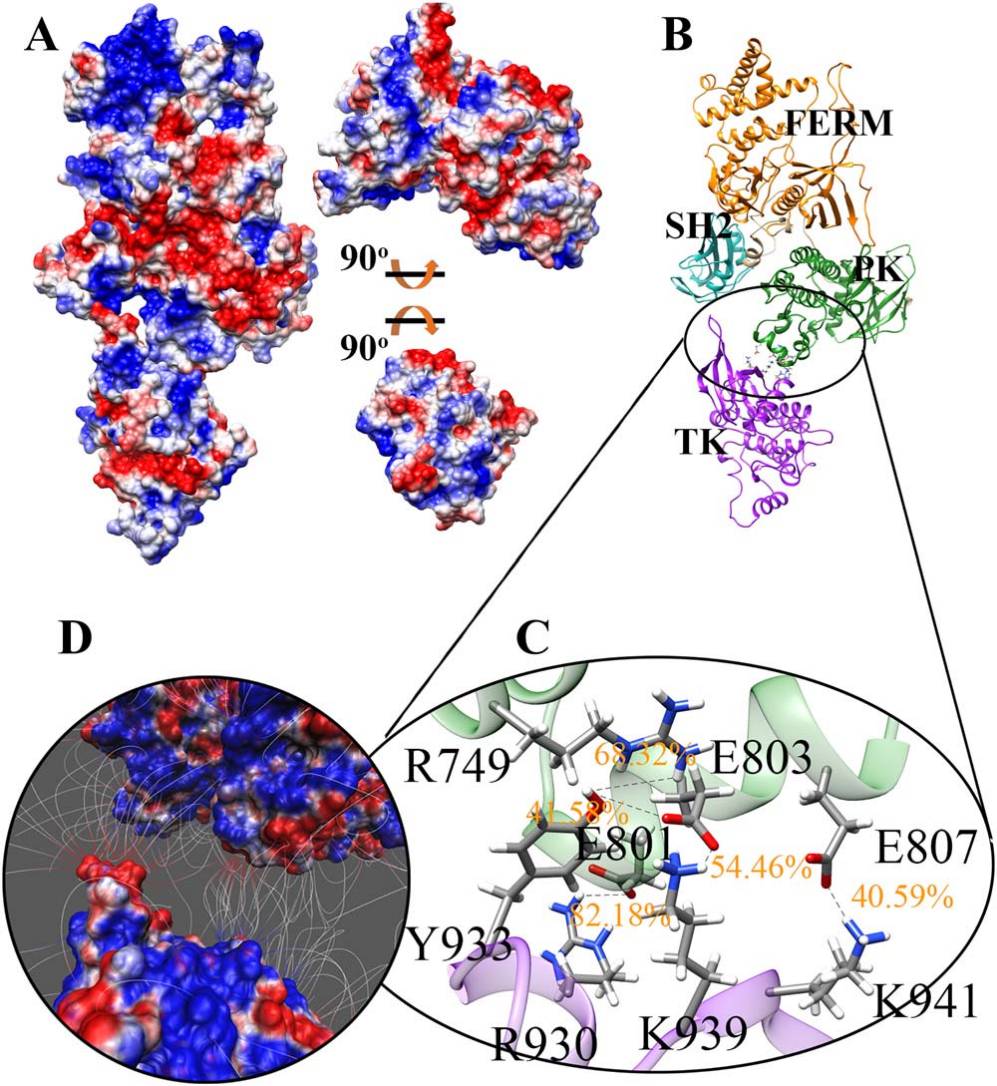
The electrostatic surface and hydrogen bonds within activated full-length JAK1 monomer. (**A**) The electrostatic surface of the full-length JAK1 structure. Positive surface (blue) and negative surface (red). Two regions (FERM-SH2-PK and TK) are rotated open 90^o^ to show the interface. (**B**) High-occupancy hydrogen bonds and structural representation of activated full-length JAK1. (**C**, **D**) High-occupancy hydrogen bonds between PK and TK are shown.

After 40 ns of MD simulations, the structure of the activated full-length JAK1 reached a stable state (Figure S2). The structures from the last 10 ns of the simulation (40–50 ns) were chosen for further analysis. In the activated structure, TK is no longer connected to FERM; instead, it interacts solely with PK (Figure 3**A** and S5). The interfaces of the FERM-SH2-PK complex and TK are displayed in Figure 3**A**. PK is positioned on the right of the FERM-SH2-PK complex, which is characterized by having mostly negative electrostatic charges. Correspondingly, the center of the TK interface is positively charged, and it is electrostatically attracted to the negatively charged PK domain surface. Hydrogen bonds were analyzed to show the individual contributions of residues (Figure 3**B-D**). R930-E801 possesses over 82.18% occupancy. Glutamic acid residues including PK domain E803 and E807 also formed high-occupancy hydrogen bonds with TK domain Y933 and K941, respectively. This shows the importance of glutamic acid residues in PK at the activated state and implicates a possible regulatory tyrosine residue Y933. Positive charges provided by R930 and K941 play a role in the electrostatic attraction to the negatively charged surface of PK, which occurs due to the presence of glutamic acid residues in the protein. The formation of hydrogen bonds between PK and TK domains is facilitated by their close proximity—typically when the distance between them is around 3.2 Å [55, 56]. This interaction contributes to the stability of the activated JAK1 structure.

### Employing Dijkstra’s method to determine the smoothest path of TK transition from inactive to active JAK1 enzyme

**Figure 4:**
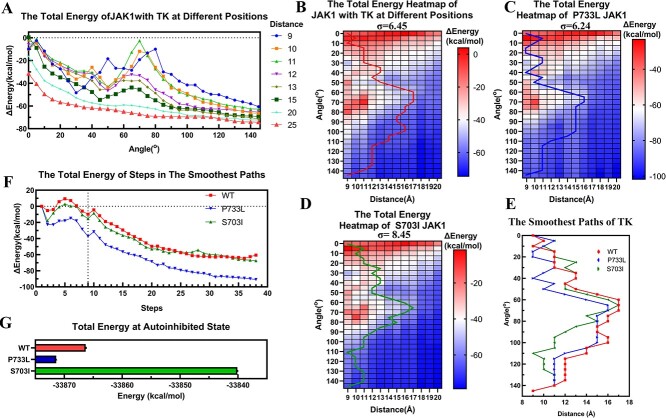
The energy analysis of the activation process of JAK1. a. The total energy of JAK1 during the rotation and separation of TK. b, c, d. The total energy heatmap of JAK1 (WT, P733L, S703I) with TK at different positions, where the black lines represent the smoothest paths for the corresponding energy heatmap. (**E**) A smoothest path comparison among WT, P733L, and S703I. (**F**) The total energy for each step along the smoothest paths for WT, P733L, and S703I JAK1. (**G**) The total energy at the autoinhibited state. The energies shown in the figure. (**B-F**) represent the relative total energy, which is the difference between the current state and the total energy in the inhibited state.

The activation of JAK1 primarily involves the dynamic transition of TK from a closed inhibited position to an open activated position. The same interface of TK facing PK at both positions suggests that JAK1 activation can be achieved by rolling TK around PK. Since efficiency is a fundamental principle for many biological processes, possible minimal paths were initially designed for TK movement. This includes separations of TK and PK by 9 Å and rotation of TK around PK by 145^o^. TK was first separated from PK by 9 Å and then rotated 145^o^ to show the total energy change (Figure 4**A**). TK encounters a high energy barrier around 60^o^-80^o^, making it difficult for the protein to continue rotating the full 145^o^. To better illustrate these energy changes, TK was further separated from PK by increments of 1 Å, up to 25 Å, to establish the point at which the energy barrier diminishes. After a 15 Å separation, the rotation did not encounter a significant energy barrier and after a 20 Å separation, there was no detectable energy barrier (Figure 4**A**). These energy calculations provide a JAK1 energy map with TK at different locations, shown in Figure 4**B**. It is evident that a region with high energy is present in the range of 912 Å and 60^o^–80^o^.

To avoid the energy barrier, we attempt to find the smoothest path for TK translocation. The smoothest path refers to the path with the lowest variance of steps. If the energy of TK translocation fluctuates markedly and frequently, the variance of is large. Here each 5^o^ rotation is considered a step. During the rotation, both separation and approach of TK can occur, leading to multiple possible paths. This means that TK moves from point M_(0,9)_ to M_(145,9)_ in Figure 4**B**. The problem of finding the minimum variance cannot be solved directly through Dijkstra’s method because the path lacks a recursive structure. To resolve this, a new contributor “${({\varDelta E}_{i,i+1}-\overline{\varDelta E})}^2$” was introduced to calculate each step’s contribution to the variance (Methods 2.3.1). This allows the creation of a recursive structure for Dijkstra’s method.

After calculations, the smoothest path is found and shown in Figure 4**B**. Clearly, during the activation, TK first separates from PK for 17 Å before overcoming the 60^o^–80^o^ rotational energy barrier. Then it approaches PK and continues rolling until reaching the activated site (Video 1). We also provide the energy heatmaps and smoothest paths of P733L and S703I JAK1 protein, shown in Figure 4**C** and **D**, respectively. The smoothest path analysis suggests that in JAK1-P733L, TK remains near PK until 55^o^ of rotation, after which it suddenly separates by about 6 Å during the next 15^o^ rotation. Subsequently, TK continues to rotate for an additional 40^o^ before abruptly approaching PK, followed by another 20 degrees of rotation, at which point TK ultimately reaches the activated site. While the energy path is smooth, the spatial pathway is not; it is interrupted by sudden separations. The ideal path should be smooth energetically and spatially—especially for the initial steps. In our models, the four domains were separated already; however, in nature, there are loops connecting several domains. The non-smooth path for TK indicates the loops between different domains are folded and unfolded frequently, which should highly energy-consuming. On the other hand, the smoothest path in space doesn’t mean the optimal path. This is because there could be clashes or energy barriers which prevent TK’s transition. Therefore, in this work, we calculated the total energy maps when TK was moved in space at variant positions and angels to find the energetically smoothest path. The smoothest path we found is the energetically smooth but the corresponding path is not spatially smooth. It means the JAK1 still have difficulty to achieve transition for JAK1-P733L. This implies the P733L mutation hampers JAK1 activation and impairs transphosphorylation. The smoothest path for TK in JAK-S703I involved an initial separation of 16 Å at a 60^o^ rotation, followed by a sudden approach over the next 50^o^ rotation. From there, it smoothly rotated to the activated state (Figure 4**D**). Compared with WT type JAK1, the initial separation combined with rotations is much slower while the final approach step is much quicker. The mutation of S703I decreases the difficulty of activating JAK1, leading to constitute activation [57]. The smoothest paths of TKs in JAK1s with and without mutations are shown in Figure 4**E**. The mutation of S703I caused spatially smooth separation and rotation initially but there is a quick approach after bypassing the high energy area. The JAK1-S703I mutation created an ‘energy stair’ for TK to navigate gradually around the high energy zone. In contrast, the JAK1-P733L mutation did not allow gradual separation of TK to bypass the high energy zone. Moreover, these mutations caused different initial total energies (Figure 4**G**). Compared with WT JAK1, the P733L mutation reduced the initial total energy, making the inhibited state more stable. Conversely, S703I increased the total energy of the inhibited state, reducing the stability of the inhibited JAK1 structure.

The energies of the smoothest paths of JAK1s are shown in Figure 4**F**. The total energy of JAK1 in the activated state is lower than that of the inhibited state. These findings indicate that the activation of JAKs could be thermodynamically spontaneous. The reason why it should be activated by cytokine signaling is necessary is because the initial separation should cross an energy barrier and enter the activated state. This energy barrier is the reason the inhibited state is also known as the ‘autoinhibited state’, and is described in many articles [4, 12, 14, 15, 19]. The VF mutation or the tyrosine phosphorylation which can activate the JAKs, could serve as the key to overcoming or diminishing the energy barrier. For the P733L mutation, which shows a low energy barrier at the initial steps but is also known to weaken transphosphorylation [20] the results could be explained by a spatial lock at step 17, where TK is at a 9 Å separation and 40^o^ rotation. The next step in the path is to be separated by 6 Å at a 15^o^ rotation, which is difficult to achieve. Thus, the JAK1-P733L structure is locked in this state where the activate site of TK is still covered.

## DISCUSSION AND CONCLUSION

Based on the previously established inhibited PK-TK dimer [15] and activated full-length mouse JAK1 homo-dimer [19], we successfully built the inhibited full-length human JAK1 and activated full-length human JAK1 monomer structures to compare the two forms as well as the transitional states of JAKs. In the inhibited state, TK is strongly locked in a cavity formed by FERM and PK domains. Glutamine residues within FERM contributed the most hydrogen bonds, while PK arginine residues contributed the most hydrogen bonds. Typically, the FERM interface facing the TK domain is negatively charged, while the TK interface facing the FERM domain is positively charged. This charge distribution is reversed in the PK-TK interfaces, with the PK domain interface being positively charged and the TK domain interface being negatively charged. In contrast, within the activated state only PK interacts with the TK domain. Consequently, fewer hydrogen bonds occur in this state contributing to a high freedom of movement for the TK domain.

Comparing the closed and open JAK1 structures, Figures 2 and 3, respectively, the interfaces of TK are distinct, suggesting that TK rotates around PK to reach the activated site. To avoid steric clashes during rotation, an initial 9 Å separation of TK is necessary for translocation. After the initial separation, the TK was further separated from PK by increments of 1 Å, up to 25 Å, and then rotated after different separations. The total energies of JAK1 with TK at different positions were calculated and inputted into a matrix suited for Dijkstra’s method where the goal is to find the energetically smoothest path of transition. This method was also applied to two pathogenic mutations, JAK1 P733L associated with a immunodeficiency phenotype [20], and the ALL linked S703I variant of JAK1 [18]. In this study we found the energetically smoothest path of P733L is not spatially fluid, specifically when bypassing a high energy zone located around 70^o^. In contrast, the JAK1 S703I variant creates an ‘energy stair’ spatially for TK to navigate around the 70° high energy zone gradually. JAK1 P733L protein is not capable of rotating around the 70^o^ high energy zone (Fig. 4**E**). These findings provide an explanation for constitutive activation of S703I and impaired autophosphorylation of P733L JAK1 proteins. Moreover, an increased initial total energy of S703I JAK1 may also contribute to hyper-activation.

In this work the electrostatic features of JAK1 in both inhibited and activated states were analyzed providing a potential pathway for TK transition to an active JAK1 structure. By comparing two distinctly pathogenic mutations, one inactivating the other activating, this study illustrates the importance of separation and rotational movements, which could be crucial for JAK activation. Furthermore, our energy analysis suggests that the inhibited state exists in a low energy state where the entire structure is stable. Presumably hyperactiving PK mutations [11, 15, 16] or tyrosine phosphorylation [11, 51] increases the energy and forces the TK domain to leave the FERM-PK cavity by rotating around PK to reach the open activated kinase state. Using JAK1 as an example, we successfully applied our comprehensive approach, using energy mapping and Dijkstra’s method, to resolve the undefined transitional movements involved in the activation of an enzymatic biomolecule. It represents the smoothest transition from an inactive to an active kinase state. This innovative approach can be applied not only to kinase proteins but also to other biomolecules that consist of multiple domains. The algorithms developed in this work are designed to study the motions between domains within multi-domain biomolecules.

**Key Points**

- The study offers an inaugural exploration of the structural transition of JAKs by combining physical and computational methods.
- The interactions between the tyrosine kinase (TK) domain and pseudo kinase domain were comprehensively analyzed by the charge distribution and hydrogen bonds for the two states: inhibited and activated states.
- The calculation of the smoothest path of TK translocation not only offers valuable insights into the activation process but also presents a novel approach to studying the significance of mutations in JAK1.

## Supplementary Material

SI_V5_bbae079

WT_bbae079

## Data Availability

The authors confirm that the data supporting the findings of this study are available within the article and its supplementary materials.
