# Supplementary material for: A novel approach to study multi-domain motions in JAK1’s activation mechanism based on energy landscape
Source: Brief Bioinform. 2024 Mar 5;25(2):bbae079. doi: 10.1093/bib/bbae079 (PMC10939344; doi:10.1093/bib/bbae079)
Supplement: SI_V5_bbae079 [file si_v5_bbae079.docx]

**Supporting Information**

**A Novel Approach to Study Multi-domain Motions in JAK1’s Activation Mechanism Based on Energy Landscape**

Shengjie Sun^1,2^, Georgialina Rodriguez^3,4^, Gaoshu Zhao^5^, Jason E Sanchez^2^, Wenhan Guo^2^, Dan Du^2^, Omar J Rodriguez Moncivais^3,4^, Dehua Hu^1^, Jing Liu^6^, Robert Arthur Kirken^3,4^, Lin Li^2,5,7^*

Correspondence to: Lin Li (e-mail: [lli5@utep.edu](mailto:lli5@utep.edu))

*^1^ Department of Biomedical Informatic, School of Life Sciences, Central South University, Changsha, China, 410083;*

*^2^Computational Science Program, The University of Texas at El Paso, 500 W University Ave, TX, 79968, USA;*

*^3^Department of Biological Sciences, The University of Texas at El Paso, 500 W University Ave, TX, 79968, USA;*

*^4^ Border Biomedical Research Center, The University of Texas at El Paso, 500 W University Ave, TX, 79968, USA;*

*^5^Google LLC, 1600 Amphitheatre Parkway Mountain View, CA 94043*

*^6^Department of Hematology, The Second Xiangya Hospital of Central South University; Molecular Biology Research Center, Center for Medical Genetics, School of Life Sciences, Central South University, Changsha 410083, China;*

*^7^Department of Physics, The University of Texas at El Paso, 500 W University Ave, TX, 79968, USA;*

**To whom correspondence should be addressed.*


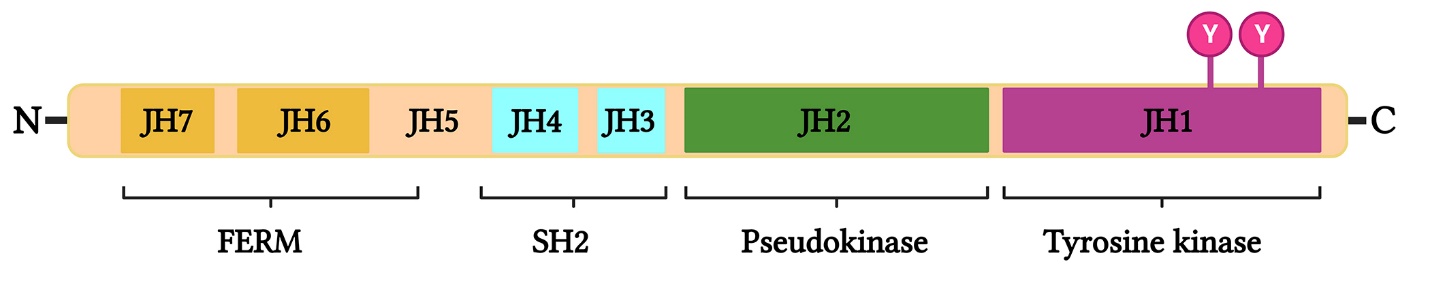


**Figure S1:** Linear structure of JAK proteins. Depiction of Janus homology (JH) domains and JAK functional domains, 4.1, Ezrin, Radixin, Moesin (FERM), Src Homology 2 (SH2), Pseudokinase (PK), and Tyrosine kinase (TK). Shown are conserved JH1 tyrosine (Y) residues involved in regulating enzymatic activity.


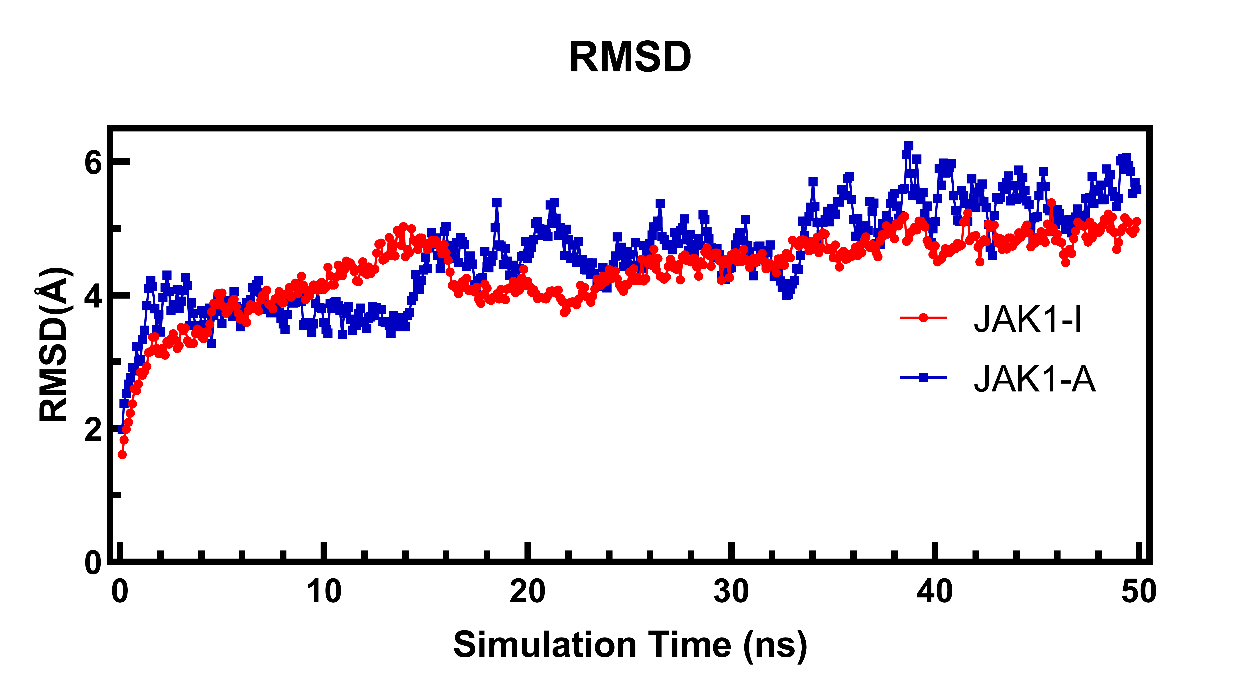


**Figure S2:** RMSD of the MD simulations of inhibited JAK1 (Red: JAK1-I) and activated JAK1(Blue: JAK1-A).


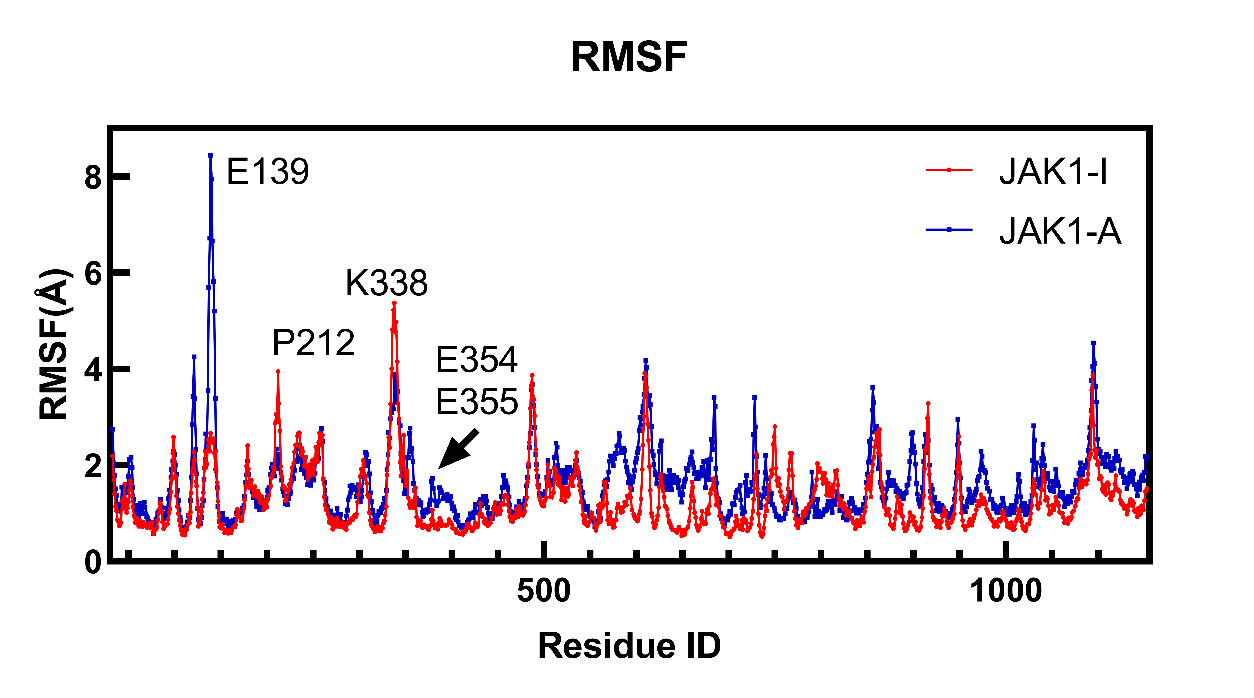


**Figure S3:** RMSF of the MD simulations of inhibited JAK1 (Red: JAK1-I) and activated JAK1(Blue: JAK1-A).


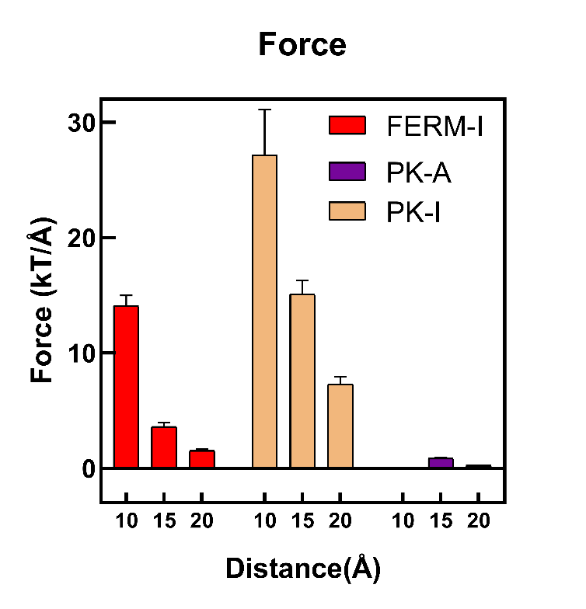


**Figure S4:** Electrostatic binding force on TK from FERM in inhibited state, (FERM-I), PK in inhibited state (PK-I) and PK in activated state (PK-A).

**Electrostatic Force Calculation Details**

The electrostatic force on the TK was calculated by DelphiForce every 0.05 ns in the last 20 ns of MD simulations (30 ns to 50 ns). The parameters for electrostatic force calculation were the same as those for potential calculation. Then the electrostatic force was decomposed into the binding component (Eq. 1) and the sliding component (Eq. 2).

$F_{Bi}=F_{i}\cdot\cos\alpha_{i}$ .……………………………………………………………… (1)

$F_{Si}=F_{i}\cdot\sin\alpha_{i}$ ………………………………….………………………….… (2)

Where the $F_{Bi}$ and $F_{Si}$ are the binding component and sliding component in the $i^{th}$ frame, respectively. $F_{i}$ is the electrostatic net force on TK at the $i^{th}$ frame. The $\alpha_{i}$ is the angle between the net force and the axis, which passed the mass centers of the PK or FERM and TK in the $i^{th}$ frame.


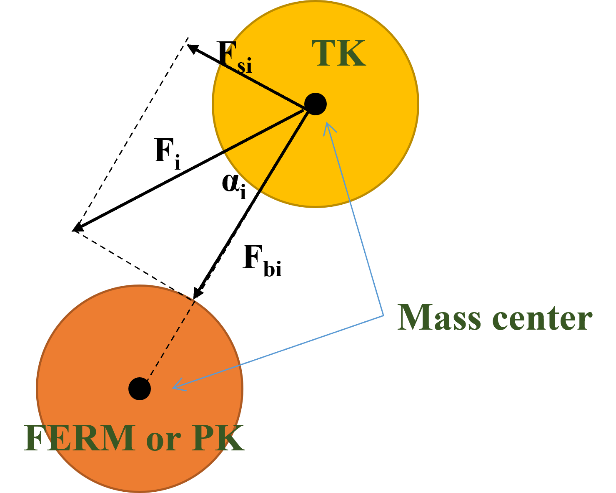


**Figure S5:** Schematic of Electrostatic Force Components.

Table S1: MD simulation parameters

| **Parameter** | **Value** |
| --- | --- |
| MD Simulation type | All-atom MD simulation |
| Simulation time | 50 ns |
| Simulation time per frame | 0.1 ns |
| Simulation time per step | 2 fs/step |
| Temperature | 310.15 K |
| Forcefield | CHARMM36m |
| Salt | 0.15M KCl |
| Size | 175 Å 175 Å × 175 Å |
| Number of water molecules | 129675 |
| Number of potassium | 135 |
| Number of chloridoid | 122 |
| Number of residues | 1141 |
| Constant temperature control | Langevin Damping: 1.0 |
| PME | Interpolation order: 6; Grid space: 1.0 |
| Cutoff | 12.0 Å |
